# Supplementary material for: Distinct subtypes of polycystic ovary syndrome with novel genetic associations: An unsupervised, phenotypic clustering analysis
Source: PLoS Med. 2020 Jun 23;17(6):e1003132. doi: 10.1371/journal.pmed.1003132 (PMC7310679; doi:10.1371/journal.pmed.1003132)
Supplement: S1 Table — The cohorts from the Hayes, Urbanek, and colleagues PCOS GWAS and corresponding numbers of samples that were included in the clustering analysis are shown by GWAS cohort, adapted from Hayes, Urbanek, and colleagues. [19]: Table 1 and Supplemental Data Tables 9 and 10. GWAS, genome-wide association study; PCOS, polycystic ovary syndrome. (DOCX) [file pmed.1003132.s002.docx]

**S1 Table. GWAS cohorts used in cluster analysis**

| **GWAS cohort** | **Source** | **Sample Size** | **Cluster analysis** | **Included** | **Excluded** |
| --- | --- | --- | --- | --- | --- |
| PCOS Family Study | US | 890 | Yes | 655 | 235 |
| PPCOSI | US | 64 | No | 0 | 64 |
| University of Chicago | US | 227 | No | 0 | 227 |
| Cedars-Sinai Medical Center | US | 130 | No | 0 | 130 |
| Massachusetts General Hospital | US | 472 | No | 0 | 472 |
| PPCOSII | US | 265 | Yes | 238 | 27 |
| University of Alabama at Birmingham | US | 193 | No | 0 | 193 |
| University of Athens | Europe | 542 | No | 0 | 542 |
| Imperial College London-University of Oxford | Europe | 222 | No | 0 | 222 |

The cohorts from the Hayes, Urbanek, and colleagues PCOS GWAS and corresponding numbers of samples that were included in the clustering analysis are shown by GWAS cohort, adapted from Hayes, Urbanek, and colleagues (2015): Table 1 and Supplemental Data Tables 9 and 10. GWAS, genome-wide association study; PCOS, polycystic ovary syndrome; PPCOS, Pregnancy in PCOS
