# Supplementary material for: Distinct subtypes of polycystic ovary syndrome with novel genetic associations: An unsupervised, phenotypic clustering analysis
Source: PLoS Med. 2020 Jun 23;17(6):e1003132. doi: 10.1371/journal.pmed.1003132 (PMC7310679; doi:10.1371/journal.pmed.1003132)
Supplement: Attachment — Submitted filename: ReviewerResponse_PLOSMed.docx [file pmed.1003132.s007.docx]

**PMEDICINE-D-19-03066 - REVISION REQUESTS FROM EDITORS AND REVIEWERS**

**Editors:**

1. *Title: Please revise your title according to PLOS Medicine's style. Your title must be nondeclarative and not a question. It should begin with main concept if possible. "Effect of" should be used only if causality can be inferred, i.e., for an RCT. Please place the study design ("A randomized controlled trial," "A retrospective study," "A modelling study," etc.) in the subtitle (ie, after a colon).*

*We suggest: “Novel genetic associations of distinct subtypes of polycystic ovary syndrome: An unsupervised hierarchical clustering analysis” or similar.*

We propose the following revised title: “Distinct subtypes of polycystic ovary syndrome with novel genetic associations: an unsupervised phenotypic clustering analysis”.

2. *Data Availability: PLOS Medicine requires that the de-identified data underlying the specific results in a published article be made available, without restrictions on access, in a public repository or as Supporting Information at the time of article publication, provided it is legal and ethical to do so. If the data are not freely available, please include an appropriate contact (web or email address) for inquiries (please note that this cannot be a study author).*

This study used data that we collected previously for our PCOS GWAS (Hayes and Urbanek *et al. Nat Commun* 6:7502, 2015) [1]. Stage 1 genotype data have been deposited in the database of Genotypes and Phenotypes (dbGaP) under the accession code phs000368.v1.p1. The following variables are provided along with the genotype data: SUBJID (de-identified), Case_Control (disease status), Sex, Age, Height, Weight, BMI, Race, Ethnicity. Subtype classifications for these subjects are provided in **S3 Table**.

This study used additional array and whole-genome sequencing data from human subjects. The majority of study subjects were enrolled prior to the implementation of the NIH Genomic Data Sharing Policy in January 25, 2015. Consequently, none of the consent forms directly addressed the broad sharing of participants’ data nor the risks associated with broad data sharing of these data. Further, consent forms limited the use of the DNA samples from PCOS cases to genetic analyses of this disorder. Therefore, individual-level data cannot be shared through NIH-designated repositories without approval of the Institutional Review Boards (IRBs) where the cohort was originally enrolled. Access to aggregate data must be limited to genetic analyses of PCOS and require approval of all relevant Institutional Review Boards. Investigators may contact the Site PIs from Hayes & Urbanek et al. [1] if they are interested in collaborating on a project that requires use of quantitative trait data.

3. *Prospective data analysis plan: Did your study have a prospective protocol or analysis plan? Please state this (either way) early in the Methods section.  If your study has an associated prospective protocol or analysis plan, please include the file as supporting information, and reference it in the Methods.*
   a. *If a prospective analysis plan (from your funding proposal, IRB or other ethics committee submission, study protocol, or other planning document written before analyzing the data) was used in designing the study, please include the relevant prospectively written document with your revised manuscript as a Supporting Information file to be published alongside your study, and cite it in the Methods section. A legend for this file should be included at the end of your manuscript.*
   b. *If no such document exists, please make sure that the Methods section transparently describes when analyses were planned, and when/why any data-driven changes to analyses took place.*
   c. *In either case, changes in the analysis—including those made in response to peer review comments—should be identified as such in the Methods section of the paper, with rationale.*

This study did not have a prospective protocol or analysis plan. The study began as an exploratory analysis to test the hypothesis that subtypes existed within the PCOS GWAS cohort. Further analyses were performed once subtypes were identified. The study unfolded chronologically as presented in the Methods. The Methods now state this (lines 162-164).

4. *Abstract: Methods and Findings: Please include some information on the population, years during which the study took place, and the study’s main outcome measures.*

The abstract has been updated for clarity and to include additional information on the study population and outcomes.

5. *Abstract: Conclusions: Line 58: The term “stable” in this sentence is unclear, please revise.*

The term was revised to “reproducible” (line 59).

6. *Author Summary: At this stage, we ask that you include a short, non-technical Author Summary of your research to make findings accessible to a wide audience that includes both scientists and non-scientists. The Author Summary should immediately follow the Abstract in your revised manuscript. This text is subject to editorial change and should be distinct from the scientific abstract. Please see our author guidelines for more information.*

An Author Summary has been added to the revised manuscript.

7. *Methods: Thank you for including your Ethics Statement on the manuscript submission form (“The study was approved by the Institutional Review Board of Northwestern University Feinberg School of Medicine (#STU00008096). All subjects gave written informed consent before study.”), can you please also include this information in the methods?*

The IRB approval information was added to the Methods on lines 166-167. The information on informed consent was retained in the Methods in lines 167-168.

8. *Methods: Please present a table of summary/demographic information for the various cohorts- the information that is in S1 Table should be moved to the main text.*

The demographic table in the **S1 Table** supplement has been moved into the main manuscript as **Table 1**. We added the family-based PCOS cohort quantitative trait data to this table, as well. The corresponding assay information has been kept as **S2 Table**. We also added a new table showing Age and BMI for each of the GWAS cohorts as **Table 2**.

9. *Results: “PCOS Subtypes”: For the percentages reported here, please present numerators and denominators.*

The numerators and denominators are now provided for each instance in the “Results: PCOS Subtypes” section.

10. *Discussion: Please present and organize the Discussion as follows: a short, clear summary of the article's findings; what the study adds to existing research and where and why the results may differ from previous research; strengths and limitations of the study; implications and next steps for research, clinical practice, and/or public policy; one-paragraph conclusion.*

Additional summary information was added to the first paragraph of the Discussion (lines 501-504), and an additional paragraph outlining implications and next steps for research, clinical practice, and/or public policy was added prior to the conclusion paragraph (lines 651-654).

11. *Figure 2: The small inset frequency histogram is difficult to read, particularly the Y axis.  Can you please provide any description of the clustering relationships among the traits displayed on the left side of the chart?*

**Figure 2** has been updated accordingly. A description of the row-based clustering has been added to the figure legend. Previously, the row-based clustering used default distance and hierarchical clustering methods, but now the row-based clustering has been calculated using the same methods used in the subject-based clustering, for consistency. This change does not alter the results, but it does slightly modify the way they are presented in **Figure 2**.

12. *Figure 5: Panel “a” is difficult to read, in particular the text of the histogram is very small (and appears to be missing an X axis label).  Please increase the size of the text.  Please define all abbreviations used in the figure legend.*

**Figure 5** has been updated for improved readability. Definitions for abbreviations used in all figures have been added to the corresponding legends.

13. *Figure 6: Please increase the size of the Y axis labels.  Please increase the size of the QQ plots, they are too small to read.*

The figure has been updated accordingly.

14. *Figure 7: The left-hand side axis is cutting off one of the CI bars for the rs55762028 risk allele.*

The x-axis in **Figure 7** was increased to include the CI bar for the rs55762028 risk allele.

15. *Figure 8, 9, 10: Please increase the text size on the graphs.*

The text on these figures have been enlarged for improved readability.

16. *Table 1: Please define abbreviations for “Chr” and “Mb” in the legend.*

Definitions for abbreviations used in all figures have been added to the corresponding legends.

17. *References: Please use square brackets for in-text citations, like this: [1].*

The citations have been updated, accordingly.

18. *Checklist: Please report your study according to the relevant guideline (STREGA or STROBE may be most appropriate), which can be found here:* [*http://www.equator-network.org/*](http://www.equator-network.org/)

*Please include the completed STROBE/STREGA checklist as Supporting Information.  When completing the checklist, please use section/paragraph numbers rather than page numbers.  Please add the following statement, or similar, to the Methods: "This study is reported as per the Strengthening the Reporting of Observational Studies in Epidemiology (STROBE) guideline (S1 Checklist)."*

The STREGA checklist has been completed and has been included as a supplement. The corresponding statement was added to the Methods (lines 164-166).

**Reviewer 1 Comments:**

1. *Abstract: The results for the unclustered 293 aren't presented? The 73 family cluster results were a surprise- should have been listed with other 2 cohorts at tope of methods.*

We have modified the Abstract to present the requested analyses.

2. *Figure 1, if possible to put reproductive, metabolic and indeterminate at the top of the heatmap, it would make it easier for the reader to interpret.*

**Figure 1** has been updated to include the subtype names within the color bar.

3. *Since total T reflect the influence of SHBG, which is different between the groups, it would be of interest to see if FAI is a better indicator, ie Fig 3*

As predicted by the reviewer, in a post-hoc assessment the FAI did differ substantially between the Reproductive and the Metabolic group (**Figure R1**). We did not use this variable for the cluster analysis because is a derived parameter based on T and SHBG levels rather than an independent variable. Only variables that were used for the cluster analysis are depicted in **Figure 3**. We believe it would be confusing to include a parameter that was not used for clustering.

**Figure R1.** Figure 3 with FAI included. Median and interquartile ranges are shown for normalized, adjusted quantitative trait distributions of genotyped PCOS cases with reproductive or metabolic subtype.

4. *Axes on Figure 8 need to be larger*

The axis labels in **Figure 8** have been enlarged.

**Reviewer 2 Comments:**

1. *First, there is no accompanying code, which makes the analyses irreproducible. My suggestion is to create a code repository (e.g., on GitHub) and upload the scripts used for clustering, GWAS and machine learning analyses. A README detailing how to access the datasets used by the code must be included.*

The scripts used to perform the GWAS used commands from off-the-shelf GWAS tools, as described in the Methods from this manuscript and our previously published GWAS [1]. The R code used to perform the clustering and subsequent family cohort classification have been uploaded to a public GitHub repository: [github.com/mdapas/PCOS_phenotype_clustering](http://github.com/mdapas/PCOS_phenotype_clustering).

2. *There is a slight disconnect between Introduction and the presented findings. Examples provided during the opening (lines 65-71) give an impression that the authors are investigating genetically heterogeneous subtypes that give rise to a convergent phenotype. However, the first step of the analysis is a phenotypic separation (i.e., divergence) of samples into clusters. This doesn't invalidate the study, but I think a minor change in the opening angle could be appropriate.*

We have clarified the first paragraph (lines 87-88). The text now explicitly refers to the convergence of different genetic etiologies under the same “clinical diagnosis” as opposed to converging under the same “phenotype”.

3. *In several places, the analysis considers projections of data onto the first k principal components. This includes outlier detection (line 190) and batch adjustment (line 200). The authors need to show that the number of components considered (two and three, respectively) captures the sufficient amount of signal in the data. The standard way of doing this is via Horn's Parallel Analysis (doi:10.1007/bf02289447) where the data is randomly permuted along each dimension, and the resulting principal component variance of the scrambled data is compared to the original.*

The use of ancestry PCs for outlier detection and population structure adjustment follows the methods used in the original published GWAS [1]. The study was designed to include only individuals of European ancestry. In the standard ancestry principal components analysis including CEU (Northern and Western European of Utah), YRI (Yoruban of Nigeria), and CHB (Han Chinese) + JPT (Japanese) reference populations, the first two principal components (PCs) reliably capture relative African and Asian ancestry. Removing individuals based on deviation from the CEU cluster in the first two PCs serves to eliminate large confounding effects due to non-European admixture. The application of this step is shown in Supplementary Figures 10-11 in Hayes et al. [1]. The same basic approach was applied by investigators for quality control of the UK Biobank [2].

The number of PCs included as covariates in genome-wide association testing varies between studies, but amongst European cohorts, the first two principal components can adequately capture population structure [3, 4]. The genomic inflation factor, λ_GC_, reflects p-value inflation due to population stratification [5]. The λ_GC_ values (<1.05) from the original GWAS indicate that the population stratification was adequately controlled [5]. In the current study, association p-values were further adjusted by λ_GC_ values for additional error control.

Most importantly, to the extent feasible, we wanted to use the same data and methods that we used in the original GWAS so that we could directly compare the subtype GWAS results to the original GWAS. We wanted to control for association test methods. Accordingly, for results that were replicated, such as the *FSHB* association with the Indeterminate phenotype, we can conclude that the replication reflects genetic concordance rather than methodological differences.

4. *I would like to see more justification for why the hierarchical clustering was collapsed to only three subtypes. To me, it seems that there's a fourth cluster within the "Indeterminate" subtype, characterized by high levels of ovarian theca cell testosterone (T) production. This is visually supported by the distinct pattern in the heatmap (Fig 2), as well as a strong alignment of T with the second principal component in both cohorts (Figs 4 and 5). If Jaccard coefficient was used to make this decision, then the authors need to also report coefficients associated with the two-cluster solution ("reproductive" and "metabolic + Indeterminate") and the four-cluster solution ("reproductive", "metabolic", "T-high Indeterminate" and "T-low Indeterminate"), highlighting that the three-cluster solution yields an inflection point.*

The selection of three clusters as opposed to two or four clusters was made for a number of reasons, both qualitative and quantitative. The primary reason three clusters were selected was because the three-cluster solution was replicated in the ungenotyped cohort, whereas the two-cluster and four-cluster solutions were not. In the genotyped cohort, the Reproductive subtype is the first outgroup, followed by a split between the Metabolic and Indeterminate subtypes, whereas in the ungenotyped cohort, the Metabolic subtype is the first outgroup, followed by a split between Reproductive and Indeterminate subtypes. Furthermore, if we were to split the Indeterminate subtype into two groups (as in a four-cluster solution), the nature of that split would be different in both cohorts. Based on these observations alone, the three-cluster solution seemed the logical choice and we did not feel that a more quantitative explanation was necessary.

Nonetheless, quantitative data also appear to support the three-cluster solution. The distinction between groups in a four-cluster solution would be much lower than between those groups in the three-cluster solution. This is indicated by the similar heights of dendrogram splits beyond n=3. In a four-cluster solution, clusters three and four would be distinguished by only one trait. If the Indeterminate group is split into "T-high Indeterminate" and "T-low Indeterminate" groups (**Figure R2**), there is complete overlap in a PC1-PC2 plot. There is greater separation between the groups along PC3, but the subtype overlap is still extensive. This higher proportion of overlap is also reflected in Silhouette plots of the three-cluster and four-cluster solutions (**Figure R3**).

If a quantitative selection is made based on Jaccard scores, the three-cluster solution is the only solution that yields multiple clusters with Jaccard scores > 0.5. The Jaccard scores for a two-sample solution in the genotyped cohort were 0.76 and 0.45; for a four-sample solution, they were 0.62, 0.49, 0.34, and 0.23.

| **A** |  | **B** |  |
| --- | --- | --- | --- |
|  | **Figure R2.** PCA plots of quantitative traits for genotyped PCOS cases with 4-cluster solution, showing A) PC2 vs. PC1 and B) PC3 vs. PC2. | | |
| **A** |  | **B** |  |
| **Figure R3**. Silhouette plots of genotyped PCOS cases with A) a 3-cluster solution and B) a 4-cluster solution. The silhouette width, s_i_, is a function of cluster overlap with the nearest cluster. Values approaching one are well-isolated from other clusters; a value of zero corresponds to a value exactly between two clusters, and a negative value means the value is more dissimilar to its own cluster than to its neighboring cluster. | | | |

5. *Please include a short statement about whether there was general agreement between the various classification methods (lines 220-225). Large discrepancy in performance may indicate that subtype classifications are not robust, and a further look at the data properties may be required to motivate the proper method choice (e.g., there may be strong nonlinear relationships between input variables that warrant the use of random forests).*

There was general agreement between the classification methods. The cross-validation results were very similar, particularly for SVM and random forest (13.6% and 13.2% error, respectively). Family cohort classification from these models yielded very similar results as well, with 86% concordance between the SVM and random forest methods. Even considering subtype classification differences between models, we believe the *DENND1A* PCA plot (**Figure 12**) clearly illustrates that the rare variant carriers tend to lie on the Reproductive side of the SHBG/LH-Ins0/BMI axis. As requested by the reviewer, the cross-validation results for each of the other methods were added to the manuscript (line 466) to show that there were not large discrepancies in performance.

6. *Lastly, I agree with the authors that the GWAS analyses may be underpowered. Given that significant signal from previous GWAS was not observed in any of the subtypes (line 392), and that there is very limited replication of hits in the Stage 2 cohort (Table 1), I feel that a number of the hits identified may actually be spurious (in particular, BMPR1B/UNC5C and CDH10). However, the authors readily admit that the GWAS findings are preliminary and provide some Hi-C data and literature-backed justification for why and how the identified variants may regulate PCOS pathways.*

The reviewer’s comment reflects a clear limitation of the study; replication of the findings is planned in future studies. Nevertheless, some of the reported associations appear quite legitimate given the existing evidence. For example, the lead *genotyped* SNP in the *PRDM2* locus was associated with the Reproductive subtype with genome-wide-significance (P_meta_=2.15×10^-9^). The *PRDM2* gene also appeared as one of the top-5 results in our family-based whole genome sequencing study [Dapas et al. JCEM 2019], as did known PCOS candidates *DENND1A*, *C9orf3*, and *BMP6*. Further, there is functional evidence supporting *PRDM2* as a candidate gene for PCOS since it is as an estrogen receptor co-activator expressed predominantly in the pituitary and ovary. The most strongly associated genotyped SNP in the *BMPR1B* locus (rs10516957) was also genome-wide significant (P_meta_=1.46×10^-8^). The genotyped SNP was nominally associated with the reproductive subtype in both the Stage 1 (OR=2.42, 1.66-3.52 95%CI, P_1_=6.72×10^−6^) and Stage 2 (OR=2.40, 1.51-3.82 95%CI, P_2_=4.7×10^−4^) analyses with nearly identical effect sizes. Further, the *BMPR1B* gene is a highly plausible candidate for PCOS based on its known biological function as a type-I AMH receptor. Additionally, while none of the previous GWAS loci reached genome-wide significance in the Reproductive and Metabolic subtypes, the *FSHB* locus was replicated in the Indeterminate cohort.

The *CDH10* and *IQCA1* findings were more tentative because of the limited association signals with only imputed SNPs. However, we did apply a number of filters to our genotype data to reduce the probability of false positives. Ancestry was controlled and adjusted for, and SNPs were filtered according to population minor allele frequency (MAF ≥0.01), Hardy-Weinberg equilibrium (p ≥1×10^-6^), call rate (≥0.99), Mendelian concordance, and duplicate sample concordance. In addition, we applied a minor allele count threshold >5 to prevent false positives due to regression modeling errors.

7. *Table S1 provides only the summary statistics. In line with the earlier comment about reproducibility, it would be helpful to include detailed instructions on how the raw data was accessed.*

The raw data comes from a large PCOS cohort we recruited and phenotyped for genetic analyses of PCOS as described in our 2015 PCOS GWAS study [1]. We did not access any external data sources. We had IRB approval to use the data for this application, and its availability is limited by the conditions outlined in our Data Availability summary (lines 681-701).

8. *Please provide reasoning behind the r^2 threshold of 0.8 on line 194. It seems a bit high given the sample size.*

The original GWAS [1] used a conservative allelic *r*^2^ threshold of 0.9 (IMPUTE2 on the Phase1 1000 Genomes reference panel) to remove questionably imputed SNPs. In an effort to improve imputation quality, we chose to use a more modern imputation method and panel in this study (Minimac3 on the Phase3 1000 Genomes reference panel). As noted by the reviewer, a lower imputation quality threshold would increase the number of variants considered in our study and, therefore, could increase our power to detect associations, which would be preferred given the limited sample sizes of the tested subtypes. However, we also wanted to tightly control for type I error, and imputation accuracy degrades rapidly for SNPs with lower MAFs [6-8]. Thus, we did not want to use a less stringent *r*^2^ threshold because our study included low-frequency variants (MAF = 0.01-0.05). Iglesias et al. [9] assessed imputation performance using Minimac3 and 1000 Genomes data. They demonstrated that imputation accuracy for low frequency variants decreased substantially for *r*^2^ < 0.8. Accordingly, we applied an imputation threshold of 0.8. We have added the Iglesias et al [9] reference to the main text (line 241).

9. *Double-check the significance of FSH in Fig3. Visually, it seems the median of each group falls within the interquartile range of the other group. I am surprised this leads to a significant distinction, especially after Bonferroni correction.*

We verified the p-values for the unpaired Wilcoxon rank-sum tests used to compare normalized trait levels between Metabolic and Reproductive subtypes. The Bonferroni-adjusted p-value for FSH was 2.45 x 10^-8^. We also confirmed that DHEAS and T were the only two traits with Bonferroni-adjusted p-values > 0.05. The comparison was well powered (n_R_=207,n_M_=329) to detect deviations of z~0.5 (0.5 SD).

10. *Figure 3 caption: missing definition of T.*

Complete trait abbreviation definitions have been added to all relevant figures.

**Reviewer 3 Comments:**

1. *The MS by Dapas et al suggests that the diagnostic rubric for "polycystic ovarian syndrome" is heterogeneous in clinical presentation and that the phenotypic heterogeneity has genetic underpinnings. The notion that PCOS is not one diagnostic category is an overdue concept. The authors of the present MS have presented data to support the notion that anovulation due to PCOS is more complex that previously appreciated. Accepting that the analytic methodology is sufficiently rigorous to support the authors' analysis and conclusions, parsing PCOS suggests that clinicians need to refine treatment approaches. Women with PCOS are poorly served by being lumped into a single diagnostic entity as doing so then suggests that all women with PCOS need similar interventions.*

We appreciate the reviewer’s comments and have included the statement that “women with PCOS are poorly served by being lumped into a single diagnostic entity as doing so then suggests that all women with PCOS need similar interventions” as a study implication in the Author Summary and Discussion (lines 81-82, 651-654).

**Reviewer 4 Comments:**

1. *The Jaccard indices, depicting that the phenotypes are really distinct, are not that high to claim that with the certainty of how it is written down in the results. In fact there is only one value that is really above 0.6 i.e. the one from the phenotype that is associated with the reproductive trait. This should be mentioned in more detail in the discussion. Although this constitutes a limitation the study is still reporting valid an interesting data and provides a novel view of how to define PCOS.*

Although the Jaccard scores demonstrate that the Reproductive and Metabolic subtypes tend to be reconstituted when bootstrapping, the scores are relatively modest. There is overlap between subtypes (**Figure R3**). Some of this overlap was likely due to the fact that all traits were fit to a normal distribution using an inverse normal transformation. The transformation was applied to reduce the likelihood that outliers would affect cluster formations. Consequently, clusters were less likely to be driven by strong effects from any single trait. The resulting Metabolic and Reproductive clusters appear to represent opposite ends of a phenotypic spectrum and align with the known pathophysiology of PCOS. The genetic association findings support the hypothesis that these are pathophysiologically discrete subtypes. In contrast, the genetic architecture of PCOS subtypes defined by the NIH and Rotterdam diagnostic criteria was generally similar suggesting that these criteria do not identify biologically discrete subtypes. The limitations of the Jaccard indices are now included in the Discussion with these additional considerations for our specific clustering approach (lines 635-650).

2. *NIH diagnosed women with PCOS constitute a subset of the whole population diagnosed according to the Rotterdam criteria. This should be mentioned in the M&M section.*

This information is now included in the Methods (lines 171-172): “Cases fulfilling the NIH criteria also meet the Rotterdam criteria for PCOS [10].”

3. *Was there any effort made to compare (in terms of general characteristics such as age, BMI etc.) the group of 893 individuals and the ones from whom not a complete set quantitative trait date were available. May be it is also worthwhile to show how many out of each cohort were selected for this analysis.*

The main reason that quantitative trait data were unavailable was differences in the endpoints assessed in cohorts contributed to our original GWAS [1] by collaborators. We have added a supplementary table, **S1 Table**, summarizing all of the GWAS cohorts and indicating the cohorts included in the cluster analysis as well as the number of cases included and excluded from those cohorts. Cases were excluded from the analysis if they did not have complete quantitative trait data available for clustering. Details of the cohorts included in the cluster analysis are included in the Methods, lines 172-182.

To address the reviewer’s comment, we compared age and BMI in the cohorts included in the cluster analysis of cases with complete quantitative trait data versus cases from the same cohort not included because of missing data. There were no significant differences in these parameters suggesting that the included cases were similar to those excluded because of missing data. This information is included in the Methods, lines 192-198.

The cohorts included in the cluster analysis were from US-based study sites. The stage 2 replication included two cohorts from Europe, in addition to US-based cohorts; neither European cohort was included in the cluster analysis because of incomplete quantitative trait data. Since European PCOS cases have a lower prevalence of obesity compared to US PCOS cases [11], it is possible that prevalence of the subtypes may differ in European cases. Accordingly, we have modified the Discussion to clarify that our study contained only US-based, European ancestry NIH PCOS, lines 625-634.

4. *I was wondering how many women were using OCP or other hormones until 3 months prior to actual phenotyping. Knowing that some effects of especially OCP's might last longer than 3 months this might have impacted on the data. For instance Testosterone levels and SHBG levels might very well be suppressed for quite some time after discontinuing OCP's. If only a limited number of women would have been using OCP's until recently this would make the data more solid than they already are. This should be mentioned too in case these data are available.*

We agree with the reviewer that the effects of contraceptive steroids on T and SHBG may last longer than 3 months. Please note that the 3 month washout period noted in the manuscript, in line 203, refers to the phenotyping of reproductively normal control women for the stage 2 replication of the GWAS [1]. Only genotypes and not quantitative trait data were included from control women in genetic association study with the subtypes. However, the same OCP washout protocol was followed for the PCOS Family Study, ungenotyped and whole-genome sequencing PCOS cohorts, the sources of the majority of the cases included in the cluster analysis [1] (**S1 Table**). All potential PCOS cases recruited for these cohorts who had been using OCPs or gonadal steroids had to have elevated total or biologically available testosterone levels and/or DHEAS levels after the OCPs or gonadal steroids were discontinued for at least 3 months in order to qualify for the study enrollment. The remaining PCOS cases were from the PPCOSII GWAS cohort [1]. PPCOSII was a randomized clinical trial of letrozole versus clomiphene citrate for infertility in PCOS [12]. The PCOS cases in this study had an OCP washout of at least 2 months prior to their baseline phenotyping visit. Since the PCOS subjects in this trial were seeking fertility, the majority were not on recent OCP therapy. Therefore, it is unlikely that recent contraceptive steroid use altered T or SHBG levels in the PCOS cases included in the cluster analysis. This information has been added to the Methods, lines 183-191.

**Additional Changes:**

- To avoid confusion, we now refer to the Stage 1 and Stage 2 GWAS data sets as “samples” and the corresponding studies that comprised these GWAS stages (e.g. PCOS Family Study or PPCOSII) are refered to as “GWAS cohorts”. We refer to the cases used in the different clustering analyses as “clustering cohorts”.
- For clarity, references to “pairwise Wilcoxon rank-sum tests” were changed to “unpaired Wilcoxon rank-sum tests”, since the tests we used assumed independent samples but “pairwise” may suggest otherwise.
- Several minor corrections were made to improve clarity throughout the text.

**References:**

1.	Hayes MG, Urbanek M, Ehrmann DA, Armstrong LL, Lee JY, Sisk R, et al. Genome-wide association of polycystic ovary syndrome implicates alterations in gonadotropin secretion in European ancestry populations. Nat Commun. 2015;6:7502. doi: 10.1038/ncomms8502. PubMed PMID: 26284813; PubMed Central PMCID: PMC4557132.

2.	Bycroft C, Freeman C, Petkova D, Band G, Elliott LT, Sharp K, et al. The UK Biobank resource with deep phenotyping and genomic data. Nature. 2018;562(7726):203-9. Epub 2018/10/12. doi: 10.1038/s41586-018-0579-z. PubMed PMID: 30305743; PubMed Central PMCID: PMCPMC6786975.

3.	Novembre J, Johnson T, Bryc K, Kutalik Z, Boyko AR, Auton A, et al. Genes mirror geography within Europe. Nature. 2008;456(7218):98-101. Epub 2008/09/02. doi: 10.1038/nature07331. PubMed PMID: 18758442; PubMed Central PMCID: PMCPMC2735096.

4.	Yu K, Wang Z, Li Q, Wacholder S, Hunter DJ, Hoover RN, et al. Population substructure and control selection in genome-wide association studies. PLoS One. 2008;3(7):e2551. Epub 2008/07/04. doi: 10.1371/journal.pone.0002551. PubMed PMID: 18596976; PubMed Central PMCID: PMCPMC2432498.

5.	Bouaziz M, Ambroise C, Guedj M. Accounting for population stratification in practice: a comparison of the main strategies dedicated to genome-wide association studies. PLoS One. 2011;6(12):e28845. Epub 2012/01/05. doi: 10.1371/journal.pone.0028845. PubMed PMID: 22216125; PubMed Central PMCID: PMCPMC3244428.

6.	Palmer C, Pe'er I. Bias Characterization in Probabilistic Genotype Data and Improved Signal Detection with Multiple Imputation. PLoS Genet. 2016;12(6):e1006091. Epub 2016/06/17. doi: 10.1371/journal.pgen.1006091. PubMed PMID: 27310603; PubMed Central PMCID: PMCPMC4910998.

7.	Wojcik GL, Fuchsberger C, Taliun D, Welch R, Martin AR, Shringarpure S, et al. Imputation-Aware Tag SNP Selection To Improve Power for Large-Scale, Multi-ethnic Association Studies. G3 (Bethesda). 2018;8(10):3255-67. Epub 2018/08/23. doi: 10.1534/g3.118.200502. PubMed PMID: 30131328; PubMed Central PMCID: PMCPMC6169386.

8.	Ullah E, Mall R, Abbas MM, Kunji K, Nato AQ, Jr., Bensmail H, et al. Comparison and assessment of family- and population-based genotype imputation methods in large pedigrees. Genome Res. 2019;29(1):125-34. Epub 2018/12/06. doi: 10.1101/gr.236315.118. PubMed PMID: 30514702; PubMed Central PMCID: PMCPMC6314157.

9.	Iglesias AI, van der Lee SJ, Bonnemaijer PWM, Hohn R, Nag A, Gharahkhani P, et al. Haplotype reference consortium panel: Practical implications of imputations with large reference panels. Hum Mutat. 2017;38(8):1025-32. Epub 2017/05/12. doi: 10.1002/humu.23247. PubMed PMID: 28493391.

10.	Diamanti-Kandarakis E, Dunaif A. Insulin resistance and the polycystic ovary syndrome revisited: an update on mechanisms and implications. Endocr Rev. 2012;33(6):981-1030. doi: 10.1210/er.2011-1034. PubMed PMID: 23065822.

11.	Essah PA, Nestler JE, Carmina E. Differences in dyslipidemia between American and Italian women with polycystic ovary syndrome. J Endocrinol Invest. 2008;31(1):35-41. Epub 2008/02/26. doi: 10.1007/BF03345564. PubMed PMID: 18296903.

12.	Legro RS, Brzyski RG, Diamond MP, Coutifaris C, Schlaff WD, Casson P, et al. Letrozole versus clomiphene for infertility in the polycystic ovary syndrome. N Engl J Med. 2014;371(2):119-29. Epub 2014/07/10. doi: 10.1056/NEJMoa1313517. PubMed PMID: 25006718; PubMed Central PMCID: PMCPMC4175743.
